# The role of A20 in the pathogenesis of lymphocytic malignancy

**DOI:** 10.1186/1475-2867-12-44

**Published:** 2012-11-07

**Authors:** Fan Zhang, Lijiang Yang, Yangqiu Li

**Affiliations:** 1 Institute of Hematology, Medical College, Jinan University, Guangzhou 510632, China; 2 College of Life science and Bio-pharmaceutical, Guangdong Pharmaceutical University, Guangzhou 510006, China; 3 Key Laboratory for Regenerative Medicine of Ministry of Education, Jinan University, Guangzhou 510632, China

**Keywords:** A20 (TNFAIP3), NF-κB, Tumor suppressor, Lymphocytic malignancy

## Abstract

Autoimmune phenomena were identified in many different cases of hematological diseases and solid tumors, which may be due to alterations in the expression and function of the NF-κB signaling pathway. Recently, a number of studies have shown that the deletion or mutation of A20, a negative regulator of NF-κB, is frequently found in lymphomas, suggesting that it may be a linker between the altered immune response and leukemogenesis. The aim of this review is to summarize current findings of the A20 biological functions and its molecular mechanism as a tumor suppressor and immune regulator. The identification of A20 mutations and deletions in lymphocytic malignancy and the predictive significance of these aberrations are also reviewed.

## Introduction

Autoimmune phenomena were identified in many different cases of hematological diseases and solid tumors in which abnormal A20 expression was characterized. Recently, a number of studies have shown that A20 deletions and mutations are frequently found in lymphomas, suggesting that it may be involved in pathogenesis [1].

A20 is also known as tumor necrosis factor-α (TNFα)-induced protein 3 (TNFAIP3), which was first discovered in 1990 by Dixit and colleagues as a cytokine-induced gene in human umbilical vein endothelial cells [2,3]. This protein was originally identified as a key regulator of inflammation signaling pathways and a negative regulator of the nuclear factor kappa B (NF-κB) activation pathway, which could attenuate the NF-κB activity triggered by signaling from several surface receptors including tumor necrosis factors receptor (TNFR), Toll-like receptor (TLR), and B cell receptor (BCR) [4].

### The A20 structure and expression features

The A20 gene is located on chromosome 6q23.3, and the cDNA sequence is 4,440 bp long with an open reading frame of 2,370 nucleotides that encodes a protein predicted to contain 790 amino acids. A20 contains seven zinc finger (ZnF) domains in its C-terminus including one that functions as an E3 ligase, while an OUT (ovarian tumor) domain is embedded in its N-terminus [2,3,5-9]. Structural studies have revealed that the fourth zinc finger (ZnF4) interacts with mono-ubiquitins and K63-linked polyubiquitin chains [9].

A20 expression may be induced in multiple organs by various stimuli including interleukin-1β(IL-1β), TNF-α, and Epstein-Barr virus (EBV) - latent membrane protein1(LMP1). Although A20 is inducible by proinflammatory cytokines in most cell types, the regulation of A20 differs in lymphocytes. Temporally restricted and tissue-specific A20 expression patterns were observed with strikingly high levels in lymphoid organs including the thymus, spleen, and gut-associated lymphoid tissue. A20 is constitutively expressed in immature and mature thymocyte subpopulations and resting peripheral T cells. T cell activation leads to the down-regulation of A20 expression in mature thymocytes and peripheral T cells. TNF does not induce A20 expression in activated T cells even though these cells express TNF receptors. Thus, although A20 has been frequently described as an inducible immediate early gene in T cells, constitutive A20 expression is the prevailing pattern in these cells. Interestingly, constitutive A20 expression is not found in resting B cells, indicating the differential regulation of this gene among the B and T lineages [4,10].

### The biological functions of A20

A20 has been reported to be a ubiquitin-editing enzyme with several functions. Although A20 was initially described as an inhibitor of TNF-induced cell death [11], subsequent studies demonstrated that A20 overexpression inhibited NF-κB activation in response to different stimuli [12,13]. Stable overexpression of A20 in a number of cell lines, such as the human breast carcinoma MCF7 cells and murine fibrosarcoma WEHI164 cells, was shown to result in partial resistance to TNF-induced apoptosis. It should be noted that inhibition of A20-mediated apoptosis has not been observed in all cell lines studied. For example, A20 overexpression in the human cervical carcinoma HeLa, lung epithelial A549, or human hepatoma HepG2 cells had no effect on apoptosis induced by the Fas receptor, lymphokine-activated killer cells, serum depletion, or oxidative stress [10]. The reason why some cell lines are protected by A20 and others are not is still unclear. However, recent studies have indicated that the most important A20 function is its function as a crucial tumor suppressor, and its deletion is closely associated with lymphomas [6].

#### NF-ÎºB inhibitory factor

**Figure 1:**
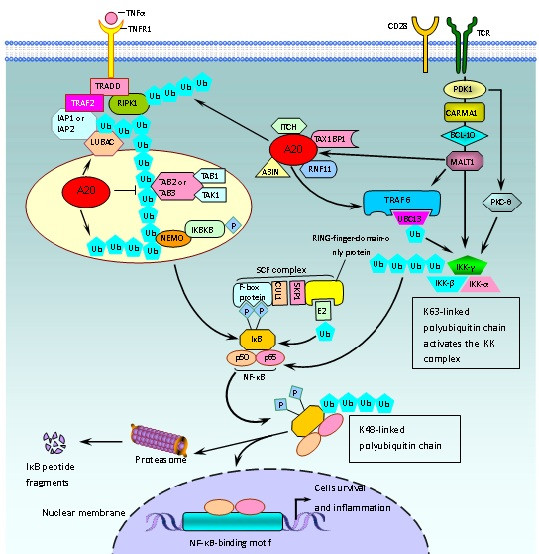
Regulation of the NF‐κB signaling related A20 protein by ubiquitylation in T cells.

The cloning and characterization of the A20 promoter revealed two NF-κB DNA binding elements, which are recognition sequences for NF-κB transcription factors. It was also found that multiple NF-κB activating stimuli induce A20 expression via NF-κB sites in the A20 promoter [14]. Therefore, A20 has been demonstrated to downregulate its own expression, and it has been proposed that A20 participates in a negative feedback loop to attenuate TNFα-induced inflammatory responses. Subsequent studies have demonstrated that A20 is also induced in many other cell types by a wide variety of other stimuli including activation of the B cell surface receptor CD40 and overexpression of HTLV-I Tax and EBV-LMP1 [15-19]. A20 overexpression was subsequently demonstrated to block the NF-κB activation mediated by TNF, IL-1, lipopolysaccharide (LPS), phorbol esters, and hydrogen peroxide in different cell types [12,13,20-23]. This blockage is most likely due to the inhibition of NF-κB activation in endothelial cells in response to proinflammatory stimuli and to an antiproliferative effect on smooth muscle cells as has been observed upon A20 overexpression *in vitro*. All of these findings suggest that A20 attenuates the activity of proximal signaling complexes at pro-inflammatory receptors [6,10]. The regulation of NF‐κB signaling as it relates to A20-mediated ubiquitylation in T cells is diagramed in Figure 1[6,24-27].

#### Regulation in innate and adaptive immunity

A20 regulates innate and adaptive immunity. This protein plays a pivotal role in the regulation of the immune response and prevents excessive NF-κB activation in response to a variety of external stimuli [28]. A20 has recently been suggested to suppress T cell activation. A20 regulates the strength and duration of the IκB kinase (IKK)/NF-κB response upon TCR/CD28 costimulation. By catalyzing the removal of K63-linked ubiquitin chains from mucosa associated lymphoid tissue 1 (MALT1), A20 prevents a sustained interaction between ubiquitinated MALT1 and the IKK complex, thus serving as a negative regulator of inducible IKK activity. Upon T cell stimulation, A20 is rapidly removed, and the paracaspase activity of MALT1, which is known to be recruited to lipid rafts after TCR stimulation, has been suggested to cleave A20 [29].

A20 loss in B cells lowers their activation threshold and enhances their proliferation and survival in a gene dose-dependent fashion. Through the expression of proinflammatory cytokines, most notably IL-6, A20-deficient B cells trigger a progressive inflammatory reaction in naïve mice, which is characterized by the expansion of myeloid cells and effector-type T and regulatory T cells [30].

Moreover, A20 also serve as a negative regulator of the Toll-like receptor, which was found to play a crucial role in controlling the maturation, cytokine production and immunostimulatory potency of dendritic cells (DCs). A20-silenced DCs demonstrated spontaneous and enhanced expression of costimulatory molecules and proinflammatory cytokines and had different effects on T cell subsets i.e., they inhibited Treg cells and hyperactivated tumor-infiltrating cytotoxic T and T helper cells, which produce IL-6 and TNF-α, and were refractory to Treg cell–mediated suppression. Hence, A20 serves as an antigen presentation attenuator for the control of the antitumor immune responses during the priming and effector phases and provides a strategy for overcoming Treg cell–mediated suppression in an antigen-specific manner, reducing the need for directly targeting Treg cells [31].

#### Tumor suppressor

There are two different A20 expression patterns in cancer cells. First, A20 overexpression was found in undifferentiated nasopharyngeal carcinoma, poorly differentiated head and neck squamous cell carcinomas (SCCs) of the skin, estrogen receptor (ER)-negative breast cancer cell lines, tamoxifen resistant ER-positive tumors, and hepatitis B virus-related hepatocellular carcinoma [32]. Our recent study also found A20 overexpression in B-ALL samples that lack an A20 mutation (unpublished), suggesting that A20 plays a role in the pathogenesis of these malignancies. Moreover, A20 overexpression has been associated with poor prognosis in ER and progesterone receptor (PR)-negative breast tumor cells; thus, it may be thought of as a new cancer biomarker [6,10,27]. Second, and more importantly, A20 is inactivated in a substantial number of lymphomas by deletion, promoter methylation, frameshift mutations, and/or nonsense mutations that result in A20 truncations or point mutations. Notably, both A20 alleles are often affected, which is a hallmark of tumor suppressor genes [6].

Increasing data support the notion that A20 is a tumor suppressor. When re-expressed in a lymphoma-derived cell line with no functional A20 alleles, wildtype but not mutant A20 resulted in cell growth suppression and apoptosis induction accompanied by the downregulation of NF-κB activation. In A20-deficient cells, the suppression of cell growth and NF-κB activity due to A20 re-expression depended, at least in part, on cell surface-receptor signaling including that of the tumor necrosis factor receptor. These findings indicate that uncontrolled NF-κB signaling caused by the loss of A20 function is involved in the pathogenesis of B cell lymphoma subsets, and they strongly suggest a tumor suppressor role for A20, the loss of which may contribute to B cell lymphoma pathogenesis by causing supra-physiological NF-κB activation, which, in turn, has oncogenic properties including inhibiting apoptosis and promoting cell proliferation [28,33].

#### Molecular mechanisms of the A20 biological functions

A20 functions as a ubiquitin-editing enzyme in the TNFR pathway by first cleaving K63-linked polyubiquitin chains on RIP1 (receptor interacting protein 1) followed by the catalysis of K48-linked chains via ZnF4, which triggers RIP1 proteasomal degradation [34]. In vitro studies conducted with recombinant A20 have indicated that A20 does not efficiently hydrolyze K63-linked polyubiquitin chains but instead has a preference for K48-linked polyubiquitin chains [35]. A20 cleaves Lys63-linked polyubiquitin chains and promotes Lys48-linked polyubiquitin chains, leading to negative regulation of TLR-mediated NF-κB and/or IRF3 (interferon regulatory factor 3) activation by regulating their substrates including TRAF6 (TNFR-Associated Factor 6), TRAF3, and RIP1 [36]. Therefore, A20 may rely on accessory proteins to provide specificity for different pathways. One such protein is TAX1BP1 (Tax1-binding protein 1), which is also known as TXBP151. TAX1BP1 is a TRAF6-binding molecule that negatively regulates TRAF6-induced NF-κB activation by cooperating with the function of A20 and the E3 ubiquitin ligase Itch, suggesting that it cooperates with A20 to inhibit cell death [37,38]. In addition, A20 associates with TXBP151 through the A20 C-terminal zinc finger-containing domain [10].

#### Regulation of A20 expression

A20 is regulated by the CARMA1–Bcl-10–MALT1(CBM) upstream signaling pathway complex, which bridges T cell antigen receptor (TCR) signaling with the canonical IKK/NF-κB pathway (Figure 1) [6,24-27]. TCR stimulation induced the recruitment of A20 and the Bcl-10 adaptor protein into the MALT1 complex, leading to MALT1-mediated A20 processing. Similarly, API2-MALT1 expression resulted in A20 cleavage. MALT1 cleaved human A20 at arginine 439 and impaired its NF-κB inhibitory function. Therefore, A20 was identified as a MALT1 substrate, emphasizing the importance of MALT1 proteolytic activity in ‘fine tuning’ T cell antigen receptor signaling [29].

Moreover, microRNA dysfunction can contribute to the constitutive activation of different signaling pathways such as the NF-κB pathway. miR-125a and miR-125b overexpression has been linked with dysfunctional hematopoiesis and aberrant immune cell responses [39-43]. miR-125a and miR-125b have been identified and characterized as regulators of A20 expression and function and, consequently, NF-κB activity. The fine-tuning of A20 levels mediated by miR-125 expression had a striking impact on the K63-linked ubiquitination of TRAF2 and RIP1, IκBα (inhibitor of nuclear factor kappa B alpha) degradation, p65 nuclear accumulation, and transcription of NF-κB target genes [44].

### A20 gene alterations in lymphocytic malignancy

Recent studies have shown that A20 is frequently inactivated by deletions and/or mutations in several lymphoma subtypes including marginal zone lymphoma (MZL), diffuse large B-cell lymphoma (DLBCL), follicular lymphoma (FL), extranodal marginal zone lymphoma (EMZL) of MALT, primary mediastinal B-cell lymphoma, and Hodgkin’s lymphoma (HL). A20 deficiency has also been found in Sezary syndrome, which is a cutaneous T cell lymphoma, particularly those characterized by constitutive NF-κB activation [1,28,33,45-47].

#### A20 deletion and mutation

There are numerous reports demonstrating different A20 mutations and deletions in lymphocytic malignancy, and the most frequent A20 alteration was found in B cell lymphoma; however, the frequency of A20 abnormalities is relatively different in different reports, while the most frequency of A20 mutations was found in B cell lymphoma. The various A20 alterations in lymphocytic malignancies are summarized in Table 1. The most frequent deletions or mutations involve exons 3, 6, and 7.

**Table 1:** A20 abnormalities in lymphocytic malignancy

**Diagnosis**	**Number of cases**	**Deletion (%)**	**Mutation (%)**	**Location**	**Reference**
cHL	36		44	OUT, ZF domain	[47]
EBV+	16		12.5	-	
EBV-	20		70	-	
NSHL	15		33.3	Exons 3, 4, and 7	[28]
other HL	27		26		[48]
MZL	32	2	19	Exons 3, 6, and 7	[46]
EMZL	11		18	Exons 3 and 7	
NMZL	9		33	Exons 3, 6, and 7	
sMZL	12		8	Exon 6	
sMZL	46	6.5	6.5	-	[49]
MALTL	30	6	17.6	C1777T; C811T; G460T/△CT1877-8	[50]
	87		21.8	Exons 3, 5, 6, 7, and 9	[28]
	64		17.2	-	[45]
	9		11	Exon 3	[1]
DLBCL	64		7.8	2	[28]
	102		38	-	[45]
GCB type	18		22.2	-	[45]
ABC type	28		50	-	[45]
GCB type	44		2.3	-	[33]
ABC type	37	32	23	-	[33]
non-GC/NC-DCBCL	20	34	22	-	[33]
MCL	35		0	-	[28]
	29		31	-	[45]
FL	52		1.9	-	[28]
	23		26	-	[45]
PMBL	14		36	-	[47]
Burkitt L	19		10.5	-	[45]
NK L	27		18.5	-	[45]
ATLL	68		10.3	-	[45]
PTCL-u	51		9.8	-	[45]
CTCL	13	46		-	[51]
ARL	33/19	18	16	-	[52]
PCNSL	32		3	-	[53]
spinal cord DLBCL	10		10	-	[53]
CLL	48	27	2	-	[54,55]
	55		0		

#### Promotor methylation

The methylation-mediated silencing of genes is an epigenetic mechanism implicated in cancer. The CpG island-associated A20 promoter was previously found in osteosarcoma cells [56]. Thus, promoter methylation is another A20 inactivation mechanism. A study by Chanudet E et al. demonstrated that A20 promoter methylation was observed in 26% (7/27) of MALT lymphoma cases including five ocular adnexal and two extra-ocular cases. Of these seven cases, six demonstrated a similar methylation pattern with prominent methylation at the 7th, 8th and 9th CpG sites. These findings are in line with the expected role of promoter methylation in the transcriptional silencing of the remaining A20 allele [50].

### The role of A20 in the pathogenesis of lymphocytic malignancy

#### The association between A20 dysfunction and lymphocytic malignancy

Recent genome-wide association studies have demonstrated a strong link between A20 polymorphisms and a collection of chronic inflammatory disorders including autoimmune diseases [5]. The phenotype of mice with full or conditional A20 deletion illustrates that A20 expression is essential for preventing chronic inflammation and autoimmune pathology. In addition, polymorphisms within the A20 genomic locus have been associated with multiple inflammatory and autoimmune disorders including systemic lupus erythematosus (SLE), rheumatoid arthritis (RA), Crohn’s disease and psoriasis [57]. Both SLE and RA are associated with a significantly increased risk of lymphoma, particularly MALT lymphoma. Moreover, MALT lymphomas of the thyroid and salivary glands also frequently arise in a background of an autoimmune disorder, and tumor cells may be directly involved in the autoimmune process [1]. These data imply that such a nonresolving inflammation, which lacks A20, is a critical component of cancer development.

#### The role of A20 inactivation in lymphocytic malignancy

The development of hematological malignancies is a multistep process that requires at least two genetic abnormalities for disease development. Increasing data regarding the role of gene alterations such as that for DNMT, TET2, IDH1/2, NPM1, ASXL1, FLT3, and EKLF for acute myeloid leukemia, STAT3 for non-GCB DLBCL, and PPP2R5C and BCL11B for T-ALL were described, and some of these genes were thought to be correlated with leukemogenesis, poor overall survival or as a prognostic or therapeutic target factor [58-62]. The novel data demonstrating the significant contribution of A20 inactivation resulting in the constitutive activation of the NF-κB pathway is considered to be associated with cancer pathogenesis; at a minimum, it plays a crucial role in the carcinogenesis of certain lymphoid malignancies. It is now clear that the genetic loss of A20 may predispose to certain lymphoid malignancies [34]. CLL with unmutated IGHV genes that demonstrate poorer survival mostly have an A20 deletion. In this case, A20 may be a biomarker and a potential target for the underlying mechanism and treatment of CLL [55].

## Summary

A20 acts as a negative feedback regulator of NF-κB activation in response to multiple stimuli and is considered a tumor suppressor. A20 dysfunction may be related to lymphocytic malignancy, and the exact A20 roles and mechanisms in the pathogenesis of lymphocytic malignancy, particularly in those with autoimmune disorders, require further characterization. Moreover, evidence has been presented suggesting that A20 and A20-binding proteins may be used as biomarkers and potential novel therapeutic targets in lymphocytic malignancies.

## Competing interests

The authors declare that they have no competing interests.

## Authors' contributions

The concept of this paper was devised by YQL. FZ, LJY and YQL contributed to the intellectual input of the paper. All authors read and approved the final manuscript.
